# Role of the Group B Antigen of *Streptococcus agalactiae*: A Peptidoglycan-Anchored Polysaccharide Involved in Cell Wall Biogenesis

**DOI:** 10.1371/journal.ppat.1002756

**Published:** 2012-06-14

**Authors:** Élise Caliot, Shaynoor Dramsi, Marie-Pierre Chapot-Chartier, Pascal Courtin, Saulius Kulakauskas, Christine Péchoux, Patrick Trieu-Cuot, Michel-Yves Mistou

**Affiliations:** 1 Institut Pasteur, Unité des Bactéries Pathogènes à Gram positif, Paris, France; 2 CNRS ERL 3526, Unité des Bactéries Pathogènes à Gram positif, Paris, France; 3 INRA, UMR1319, MICALIS, Jouy-en-Josas, France; 4 AgroParisTech, UMR MICALIS, Jouy-en-Josas, France; 5 INRA, Plate-forme MIMA2, Jouy-en-Josas, France; Children's Hospital Boston, United States of America

## Abstract

*Streptococcus agalactiae* (Group B streptococcus, GBS) is a leading cause of infections in neonates and an emerging pathogen in adults. The Lancefield Group B carbohydrate (GBC) is a peptidoglycan-anchored antigen that defines this species as a Group B Streptococcus. Despite earlier immunological and biochemical characterizations, the function of this abundant glycopolymer has never been addressed experimentally. Here, we inactivated the gene *gbcO* encoding a putative UDP-N-acetylglucosamine-1-phosphate:lipid phosphate transferase thought to catalyze the first step of GBC synthesis. Indeed, the *gbcO* mutant was unable to synthesize the GBC polymer, and displayed an important growth defect *in vitro*. Electron microscopy study of the GBC-depleted strain of *S. agalactiae* revealed a series of growth-related abnormalities: random placement of septa, defective cell division and separation processes, and aberrant cell morphology. Furthermore, vancomycin labeling and peptidoglycan structure analysis demonstrated that, in the absence of GBC, cells failed to initiate normal PG synthesis and cannot complete polymerization of the murein sacculus. Finally, the subcellular localization of the PG hydrolase PcsB, which has a critical role in cell division of streptococci, was altered in the *gbcO* mutant. Collectively, these findings show that GBC is an essential component of the cell wall of *S. agalactiae* whose function is reminiscent of that of conventional wall teichoic acids found in *Staphylococcus aureus* or *Bacillus subtilis*. Furthermore, our findings raise the possibility that GBC-like molecules play a major role in the growth of most if not all beta –hemolytic streptococci.

## Author Summary

*Streptococcus agalactiae* (Group B Streptococcus) is a leading cause of sepsis (blood infection) and meningitis (brain infection) in newborns and in adults with underlying diseases. *S. agalactiae* is a Gram-positive coccus surrounded by a thick cell wall that acts as an exoskeleton to guarantee resistance to mechanical stresses and maintenance of cell shape. Understanding the organization and the functioning of the cell wall is very important as this cellular compartment is essential to bacterial physiology and the target of many antibiotics. In this report, we have discovered the first gene *gbcO* involved in the synthesis of an abundant polysaccharide anchored to the peptidoglycan known for many years as the Group B antigen (GBC). We have constructed the first GBC-depleted strain of *S. agalactiae* (Δ*gbcO*) that displayed important growth-related defects due to mislocalization of peptidoglycan synthesis and remodeling enzymes. The phenotypes of the *ΔgbcO* mutant are similar to those observed for a Δ*tarO* mutant of *Staphylococcus aureus*, *tarO* being involved in the first step of the biosynthesis of wall teichoic acid (WTA). Hence, our results strongly suggest that GBC is the functional homolog of WTA in GBS, both being peptidoglycan-anchored glycopolymers required for the maintenance of normal growth and proper cell division. Based on genome comparisons, we postulate that GBC-like molecules with similar functions are synthesized by other streptococcal species responsible for a variety of infectious diseases in human and animals. These putative biosynthetic pathways might constitute attractive targets for the development of novel antimicrobial molecules.

## Introduction

**Figure 1:**
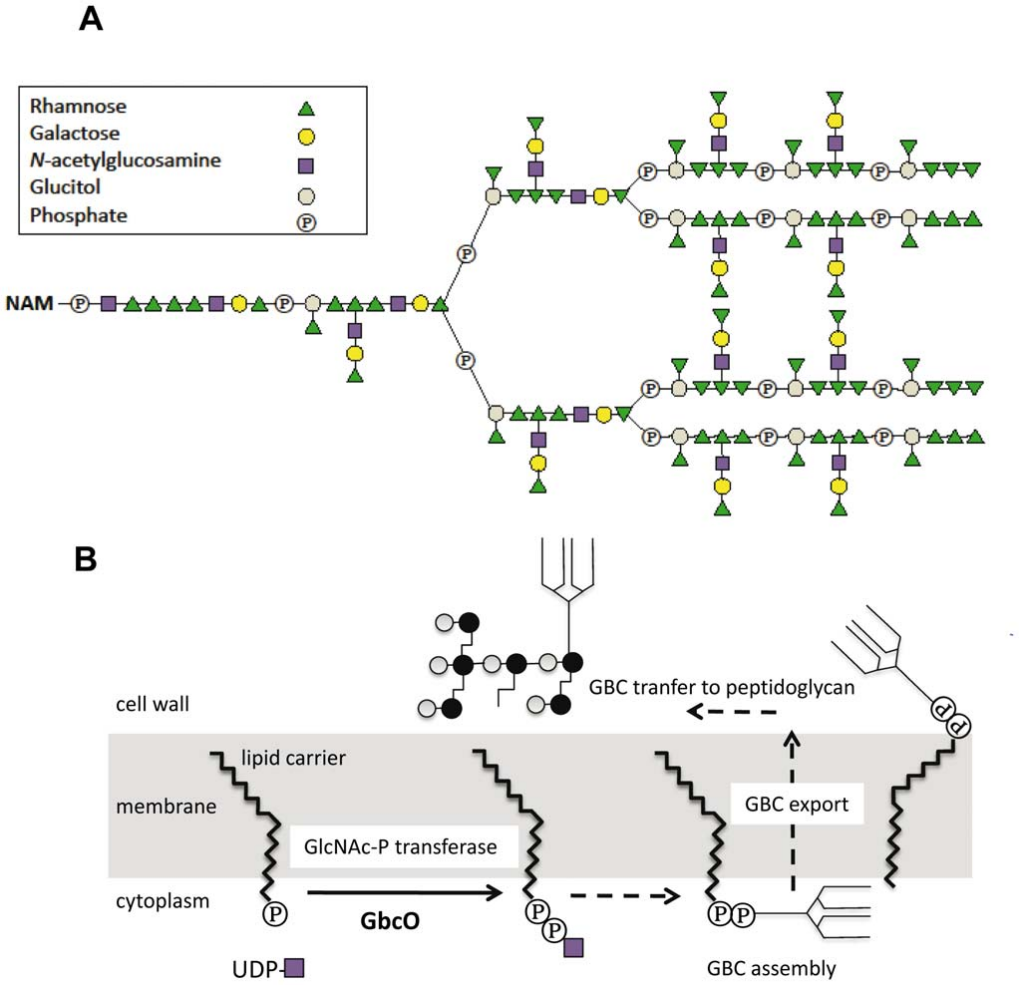
Structure of GBC and proposed scheme of GBC synthesis. (**A**) The multiantennary GBC is shown linked to an N-acetyl muramic (NAM) moiety, a component of PG. (**B**) The figure depicts the first steps of GBC synthesis where GbcO is proposed to catalyze the transfer of UDP-GlcNAc to a lipid phosphate carrier.

*Streptococcus agalactiae* was first recognized as a veterinary pathogen causing mastitis in cattle and later as a human pathogen responsible for severe neonatal infections [1]–[4]. While it remains a major cause of morbidity and mortality in infants, *S. agalactiae* is a human commensal that colonizes the rectal and the vaginal mucosa of 15–30% of women [3], [5]. Rebecca Lancefield originally defined two cell wall carbohydrate antigens in *S. agalactiae*: the group B-specific antigen (GBC) common to all strains and the capsular antigen which currently defines 10 different serotypes (Ia, Ib, II to IX) [6]. The complex multiantennary structure of GBC based on the arrangement of four different oligosaccharides (rhamnose, galactose, N-acetylglucosamine, and glucitol) (**Figure 1A**) was solved in a series of seminal studies at the end of the 80's [7], [8]. More recently, the capsular polysaccharide and the group B carbohydrate were shown to be covalently bound to the peptidoglycan (PG) at separate sites, i.e. to N-acetylglucosamine and N-acetylmuramic acid respectively [9]. Based on an initial prediction made from genome analysis [10], a comprehensive *in silico* reconstruction of the biosynthetic pathway of GBC was recently proposed by Sutcliffe and coworkers [10], [11]. Despite the importance of GBC in medical microbiology, the biological role of this surface polysaccharide is unknown and the genetic basis of its biosynthesis was not addressed experimentally.

In Gram-positive bacteria, the cell envelope contains carbohydrate-based anionic polymers that play important role in extracellular interactions and as scaffolds for enzymes required in cell wall metabolism [12], [13]. The two major classes of anionic polymers are the lipoteichoic acids (LTA) associated to the plasma membrane and the wall teichoic acids (WTA) covalently anchored to the PG. WTA, that have been extensively studied in *Bacillus subtilis* and *Staphylococcus aureus*, were reported to be essential for proper cell division and morphology [14], [15]. WTA are made of linear chains of glycerol-phosphate in *B. subtilis*, or ribitol-phosphate in *S. aureus*, which are attached to the C-6 of the MurNAc residues of PG *via* a sugar-containing linkage unit [16]. Interestingly, there is no report of the presence of similar type of polyAlditol phosphate WTA (pAdoP-WTA) in the cell wall of streptococci including *S. agalactiae* [9], [17], [18]. Consistently, the analysis of the genome of *S. agalactiae* did not reveal the presence of genes orthologous to *tagABDEF* or *tarABFKL* involved in the biosynthesis of pAdoP-WTA in *B. subtilis* or *S. aureus*, respectively. However, we identified a *tagO*/*tarO* orthologous gene in all sequenced GBS strains (*gbs0136* in the strain NEM316) that could encode an enzyme catalyzing the transfer of N-acetylglucosamine-1-phosphate to bactoprenyl phosphate, i.e. the first step in the WTA biosynthetic pathway [15], [19]. The presence of a gene encoding a TarO ortholog in GBS genome (thereafter named *gbcO*) suggests that a cell wall-linked glycoconjugate is synthesized in GBS. In the absence of a recognizable pAdoP-WTA biosynthetic pathway in *S. agalactiae*, and as already proposed by Sutcliffe and coworkers in their bioinformatic analysis [11], we hypothesized a link between *gbcO* and the Group B antigen biosynthesis.

Here, we report the detailed analysis of a *gbcO* deletion mutant of *S. agalactiae* as the first GBC-depleted strain of *S. agalactiae*. The Δ*gbcO* mutant displayed aberrant cell morphology and major cell division defects due to i) an abnormal distribution of the sites of active PG synthesis, ii) a marked decrease of peptidoglycan reticulation, and iii) an improper localization of PcsB, a major streptococcal PG hydrolase. In conclusion, our results demonstrate that GBC plays an essential role in the streptococcal morphology and bacterial growth and strongly suggest that this PG-anchored rhamnose-rich anionic polysaccharide should be considered as a functional homolog of the conventional WTA characterized in *S. aureus* and *B. subtilis*.

## Results/Discussion

### GbcO is required for biosynthesis of cell wall-anchored GBC antigen

**Figure 2:**
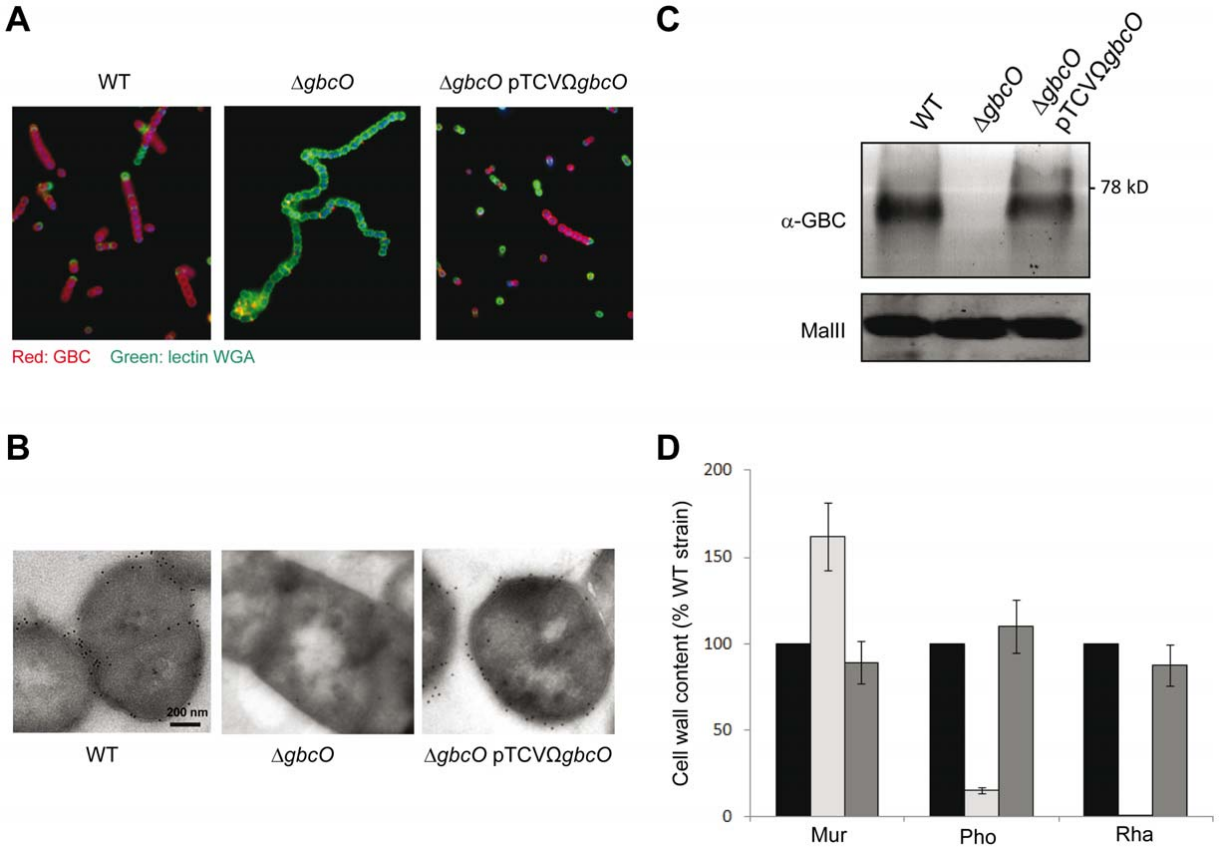
GbcO is required for GBC synthesis. (**A**) IFM of bacteria harvested in stationary phase and labeled with anti-GBC serum and Wheat Germ Agglutinin lectin to detect GBC (red) or PG (green), respectively. The representative views show the GBC antigen exposed at the surface of NEM316 WT and complemented (Δ*gbcO*pTCVΩ*gbcO*) strains but not at the surface of the *ΔgbcO* mutant. (**B**) Immunoelectron microscopy (IEM) of NEM316 WT, Δ*gbcO* mutant and complemented (Δ*gbcO* pTCVΩ*gbcO*) strains. The subcellular localization of GBC was analyzed using IEM on thin sections (<100 nm) of frozen cells; labelled with anti-GBC serum and revealed with colloidal gold particles (black dots). Black dots are clearly visible on the periphery and septa of cells of WT and complemented strains; no labeling can be detected with the *ΔgbcO* strain. (**C**) Immunodetection of GBC in mutanolysin cell wall extracts obtained from cultures harvested in stationary phase. Cell wall extracts were treated with pronase, separated on SDS-PAGE, transferred on nitrocellulose and membrane incubated with anti-GBC serum. In this experiment, the GBC-associated signal appeared as a single band that was undetectable in *ΔgbcO* cell wall extracts. As a loading control, cell wall extracts before pronase treatment were probed with the biotinylated MalII lectin. (**D**) Analysis of muramic acid, phosphate, and rhamnose content of the cell wall of WT (black bars), *ΔgbcO* (light gray bars), and complemented (dark gray bars) strains harvested in stationary phase (see **Text S1** in supporting information). For each compound the GC-MS analysis result is presented as a percentage of the WT value. Rhamnose, the main GBC sugar, was not detected in the cell wall of the *ΔgbcO* strain. Error bars represent ± S.E. of two independent experiments.

The *gbcO* gene of the *S. agalactiae* NEM316 wild-type (WT) strain, which encodes a TagO/TarO ortholog (**Figure S1**), was inactivated to investigate its role in GBC biosynthesis (**Figure 1B**). As all attempts to construct an in-frame deletion mutant of *gbcO* were unsuccessful, we deleted it by allelic replacement with a promoter- and terminator-less kanamycin marker [20], [21]. Thanks to the use of this positive selection system, the strain NEM2772 (Δ*gbcO*) bearing an inactivated *gbcO* gene was isolated and a complemented strain (*ΔgbcO*pTCVΩ*gbcO*) was constructed by re-introducing a functional *gbcO* gene cloned onto a low-copy-number plasmid. To validate the role of *gbcO* in the biosynthesis of GBC, *S. agalactiae* NEM316 WT, the isogenic *ΔgbcO* mutant, and the complemented strains were probed with a rabbit anti-GBC polyclonal antibody [22]. Immunofluorescence microscopy (IFM) analysis using Wheat Germ Agglutinin to label the whole bacteria and specific GBC antiserum revealed the presence of GBC at the surface of WT strain and its absence in the *ΔgbcO* mutant (**Figure 2A**). This defect was complemented in the *ΔgbcO* mutant transformed with the pTCVΩ*gbcO* plasmid. This result demonstrates that *gbcO* restores the exposure of GBC at the bacterial surface. Quantification of the immunofluorescence data by flow cytometry using simple immunolabeling with the anti-GBC serum indicated no significant differences between wild-type and complemented strains (data not shown).

The essential role of GbcO in GBC synthesis was confirmed by immunogold transmission electron microscopy (TEM) experiments (**Figure 2B**). The immunolabeling electron micrographs show the presence of GBC (black dots) at the periphery and septa of cells of WT and complemented strains while no gold particles were detected on *gbcO* mutant cells. To demonstrate that the immuno-reactive molecule was associated to PG as it is expected for the GBC surface antigen [9] and that the anti-GBC serum does not cross-react with proteins, mutanolysin extracts were treated with pronase, separated on SDS-PAGE, transferred on nitrocellulose, and probed with the anti-GBC serum. As shown in **Figure 2C**, the GBC signal appears as a single band in WT and Δ*gbcO*pTCVΩ*gbcO* extracts which was absent in the Δ*gbcO* sample. This series of experiments provided immunological evidences that GBC was absent from the surface of the Δ*gbcO* mutant, restored in the complemented strain, and that the GBC antiserum specifically recognizes a non-proteinaceous material associated to the PG.

As mentioned above (see **Figure 1A**), the GBC molecule has a high phosphate and rhamnose content and is likely the major source of these two compounds in *S. agalactiae* envelope. Thus, to confirm the absence of GBC in the *ΔgbcO* mutant by an alternative approach, we performed a quantitative analysis of rhamnose and phosphate present in the insoluble (PG-associated) cell wall fractions of NEM316 WT, Δ*gbcO*, and complemented strains. We also measured in the same samples the muramic acid content originating from the PG glycan chain. The most striking result of these analyses was the disappearance of rhamnose in the *ΔgbcO* cell wall whereas the WT level was restored in the complemented strain (**Figure 2D**). In the *ΔgbcO* sample, we also observed a strong decrease (85%) in the phosphate content, showing that GBC is a major phosphate source in this cellular compartment. As the same mass was analyzed for the three strains, we determined that the absence of GBC in the Δ*gbcO* cell wall increased the relative amount of peptidoglycan (measured as an increased in muramic acid) in the analyzed sample. The increase of muramic acid in NEM2772 (Δ*gbcO*) sample showed that GBC is a major constituent of the cell wall of *S. agalactiae* that represents more than 60% of the PG dry weight in WT cell wall, a value in good agreement with previous analysis [17]. Interestingly, these values are in good accordance with the amount of WTA present in the cell wall of *B. subtilis* or *S. aureus* [15]. Taken together, these immunological and biochemical data strongly suggested that GbcO catalyzes the first enzymatic step of GBC synthesis.

### GbcO is an UDP-GlcNAC:lipid phosphate transferase and a functional homolog of TarO from *S. aureus*

**Figure 3:**
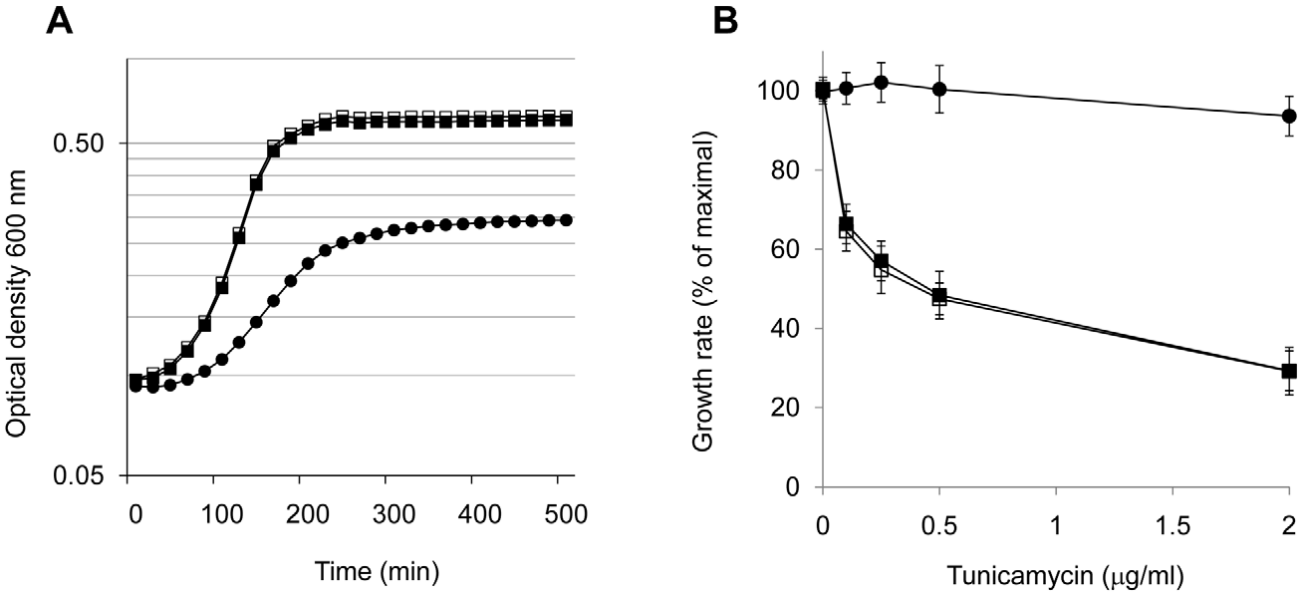
Decreased growth rate and lack of tunicamycin sensitivity of *ΔgbcO* mutant. (**A**) Growth curves of NEM316 WT (solid squares), Δ*gbcO* mutant (circles) and Δ*gbcO*pTCVΩ*gbcO* (empty squares) strains. Cultures were performed in TH medium without antibiotics at 37°C in 96 wells plates in triplicate. Optical densities were recorded at 600 nm in a Tecan M200 apparatus with 5 sec agitation before measure. Average values of a typical experiment are presented. (**B**) Effect of various concentrations of tunicamycin on the growth rate of WT (solid squares), *ΔgbcO* (black circles) and Δ*gbcO*pTCVΩ*gbcO* (empty squares) strains. Tunicamycin, a general inhibitor of UDP-GlcNAc:lipid phosphate carrier transferase activities, inhibits the growth of WT and complemented strains but not that of *ΔgbcO* mutant suggesting that GbcO carries this activity. Experiments were performed in triplicate and results are reported as a percentage of the growth rate in absence of tunicamycin. Error bars represent ± S.E. of triplicate experiments.

The GBS *ΔgbcO* mutant displayed a slower exponential growth rate constant in TH broth as compared to NEM316 WT (**Figure 3A**). The generation times were estimated to be 48 min for WT strain *vs* 138 min for the *ΔgbcO* mutant, while that of the complemented strain was restored to the WT value. To determine whether GbcO truly encodes an UDP-GlcNAC:lipid phosphate transferase, we measured the growth of the three strains in the presence of tunicamycin, a specific inhibitor of UDP-GlcNAc to lipid-phosphate carriers transferases [EC:2.7.8.-] [23], [24]. Our underlying hypothesis being that tunicamycin should inhibit the growth of strains expressing GbcO but not that of GbcO-defective strain. Maximal growth rates were measured in TH broth in the presence of increasing concentrations of tunicamycin. We consistently observed that the relative growth rates of NEM316 WT and *ΔgbcO* complemented strains decreased by up to 70% whereas that of the *ΔgbcO* mutant remained unaffected in the tested antibiotic concentration range (**Figure 3B**). Tunicamycin was recently used to inhibit WTA synthesis in *S. aureus* and the impact of the drug on growth rate was in the same order of magnitude as that observed for *S. agalactiae* [24]. Microscopic examination of NEM316 WT cells grown in the presence of tunicamycin showed morphological changes similar to those resulting from deletion of *gbcO* (**Figure S2**). These results are fully compatible with GbcO acting as an UDP-GlcNAC:lipid phosphate transferase involved in the first step of GBC synthesis.

**Figure 4:**
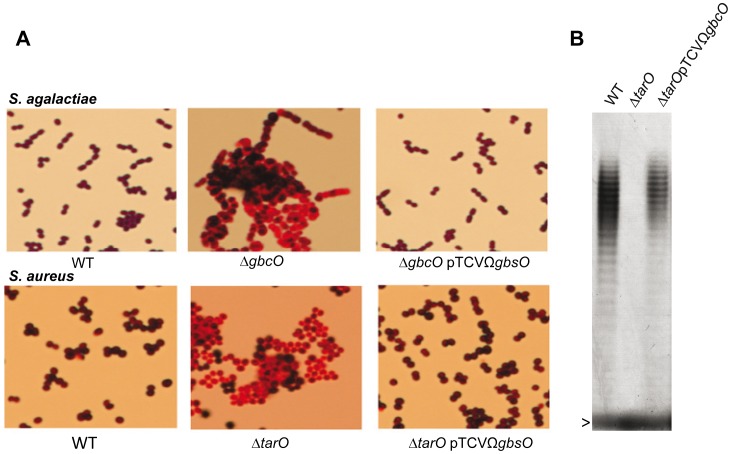
GbcO functionally complement TarO of *S. aureus*. (**A**) *S. agalactiae ΔgbcO* or *S. aureus ΔtarO* strains does not take Gram staining. In both species, the Gram staining and morphological phenotypes are restored by introduction of the plasmid pTCVΩ*gbcO* carrying a functional *S. agalactiae gbcO* gene. (**B**) PAGE analysis of WTA extracted from *S. aureus* visualized with the alcyan blue-silver staining protocol. The gel shows the production of WTA in RN4220WT (first lane), the absence of WTA in the *S. aureus ΔtarO* strain (second lane) and the restoration of the WTA synthesis when the *tarO* deficiency is complemented in trans with the streptococcal *gbcO* gene (third lane). The arrowhead indicates the bromophenol blue migration front.

The sequence homology between the streptococcal GbcO and staphylococcal TarO proteins suggests that they catalyze the same enzymatic reaction (**Figure S1**). To ascertain this hypothesis, the complementing plasmid pTCVΩ*gbcO* was introduced into the *S. aureus* RN4220Δ*tarO* mutant. This complementation experiment revealed that the morphological and Gram staining defects of the *S. aureus* Δ*tarO* mutant were corrected by expression of the streptococcal *gbcO* gene (**Figure 4A**). To prove that the heterologous complementation of Δ*tarO* was fully functional, we performed the extraction and analysis of WTA in the three staphylococcal strains following established protocols [25], [26]. The results shown in **Figure 4B** unambiguously demonstrate WTA production in RN4220 WT and complemented Δ*tarO*pTCVΩ*gbcO*, but not in RN4220Δ*tarO* mutant. The fact that GbcO can functionally complement TarO provides further support for the hypothesis that GbcO is an UDP-GlcNAC:lipid phosphate transferase. These results demonstrated that, although the cell wall anionic polymers GBC and polyribitol WTA are structurally and genetically unrelated, the first step of their synthesis involves the same enzymatic reaction.

Surprisingly, the Gram staining of *S. agalactiae ΔgbcO* like that of *S. aureus* Δ*tarO* was abnormal, a phenotype that was corrected in the complemented strains (**Figure 4A**). This serendipitous observation showed that GBC and WTA, are directly or indirectly involved in the retention of the crystal violet-iodine complex in the bacterial cytoplasm and suggests that the presence of a charged glycopolymer in the cell wall of Gram-positive bacteria rather than the PG thickness is a major determinant of the Gram staining procedure.

### Cell morphology, septa location, and cell separation are affected in the GBC-depleted mutant

**Figure 5:**
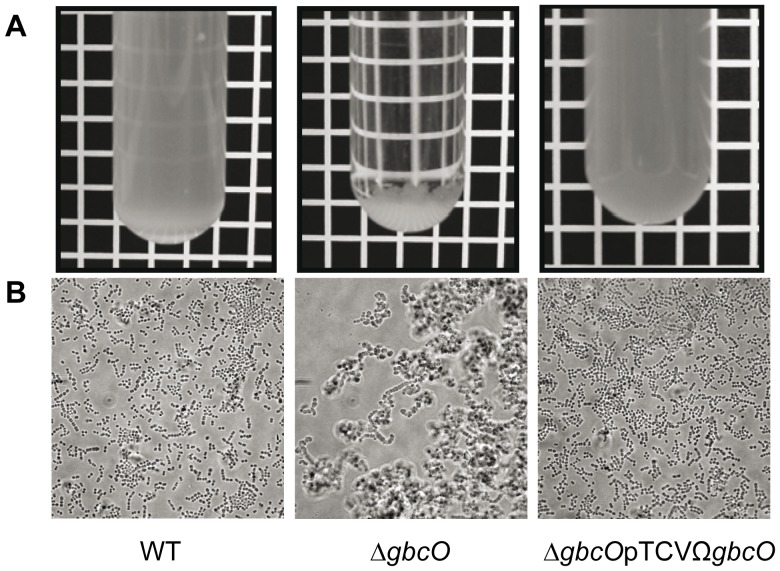
Flocculation and aggregation phenotypes of *ΔgbcO* mutant. (**A**) Overnight cultures showing the non-flocculating NEM316 WT and complemented strain (*ΔgbcO*pTCVΩ*gbcO*) and the flocculating *ΔgbcO* mutant. (**B**) Phase contrast views illustrating the morphological switch from small individual chains to large bacterial clusters characteristic of *ΔgbcO* mutant (Scale bar, 5 µm).

**Figure 6:**
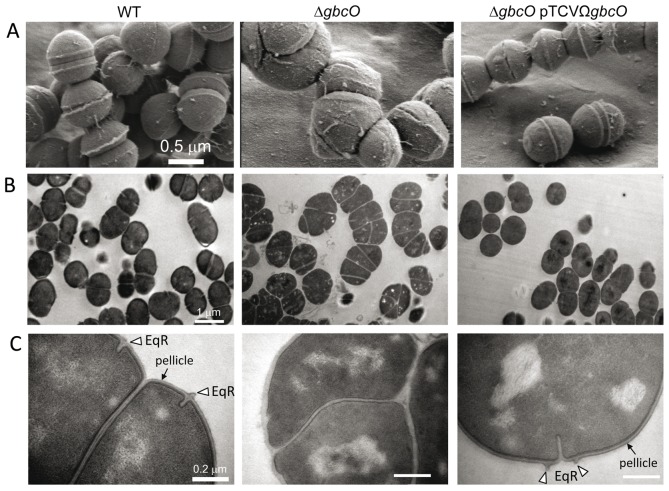
Electron microscopy imaging of NEM316 WT, *ΔgbcO* mutant, and complemented strains. Bacteria were harvested in mid-log phase (OD_600 nm_ = 0.5), fixed, and prepared as described in Supporting Materials and Methods (see **Text S1**) (**A**) Representative views of scanning electron microcopy analysis illustrating the morphological alterations (size, form, and cell division abnormalities) due to *gbcO* inactivation. (**B, C**) Transmission electron microscopy views of uranyl acetate stained thin cryosections at two magnifications (see scale bars). The presence of the pellicle (electron dense outer layer) at the surface of WT and complemented strains observed at the higher magnification is highlighted with black arrows. An open triangle depicts the equatorial ring (EqR), a zone of active peptidoglycan synthesis seen in almost all WT and complemented cells but absent in the *ΔgbcO* mutant cells.

In standing cultures, the *ΔgbcO* mutant strain tends to flocculate rapidly (**Figure 5A**) and phase contrast microscopy observations revealed the presence of large cellular aggregates instead of small typical chains of ovococci (**Figure 5B**). A careful examination of *ΔgbcO* cell clusters (**Figure 5B**) suggested that they each originated from the folding of a unique chain. To confirm this observation, we followed the growth of GBS *ΔgbcO* mutant cells in time-lapse experiments under light microscopy. This experiment revealed that clusters of *ΔgbcO* mutant cells arose from the growth of a single chain that does not break whereas the WT strain forms short individual chains (see **Video S1** for NEM316 WT and **Video S2** for *ΔgbcO* in supporting information). This observation indicated that the cell separation process of *S. agalactiae* was strongly altered in the absence of GBC. The cell and chain morphology of WT, mutant, and complemented strains were then examined by scanning electron microscopy (SEM) and transmission electron microscopy (TEM). As expected, NEM316 WT and complemented strains cells displayed regular size and were assembled in typical ovococci chains, with septa formed in successive parallel planes perpendicular to the chain axis (see **Figure 6A, 6B** and **Figure S3**) [27], [28]. By contrast, no regular pattern of division can be observed for the *ΔgbcO* mutant: cells were heterogeneous both in size and form and the septa localization seemed to occur randomly (**Figure 6A 6B** **; Figure S3**). Furthermore, in the mutant strain, the septation process was incomplete and cells were poorly individualized explaining the abnormal growth mode observed in time-lapse experiments.

As in many streptococcacae (25, 26), WT and complemented strains displayed a peripheral electron dense zone (mean thickness 6.03±1.07 nm) that was not observed in *ΔgbcO* cells (**see arrows in** **Figure 6C**). This structure must not be confused with the polysaccharide capsule of *S. agalactiae* that cannot be detected by the conventional heavy metal staining procedures used here. This cell wall structure, the function of which is unknown was named “pellicle” in *Lactococcus lactis* and, although its composition was not formally established, its presence correlated with the synthesis of a surface polysaccharide [29]. It thus appears that, as shown in *L. lactis*, depletion of a cell wall associated polysaccharide in *S. agalactiae* led to the disappearance of the pellicle.

Lastly, as observed with all streptococci, GBS WT and *ΔgbcO* complemented strains exhibited equatorial rings (EqR), i.e. a cell wall outgrowth associated to an underlying membrane invagination (**see triangles in** **Figure 4C**) where cell division (Fts proteins) and PG synthesis (penicillin-binding proteins) machineries are assembled to prime the assembly of new wall [27]. This structure was never observed in *ΔgbcO* mutant suggesting that the PG structure could be altered in the absence of GBC.

### Cell wall-anchored GBC is required for normal PG structure and correct positioning of PG synthesis sites

**Figure 7:**
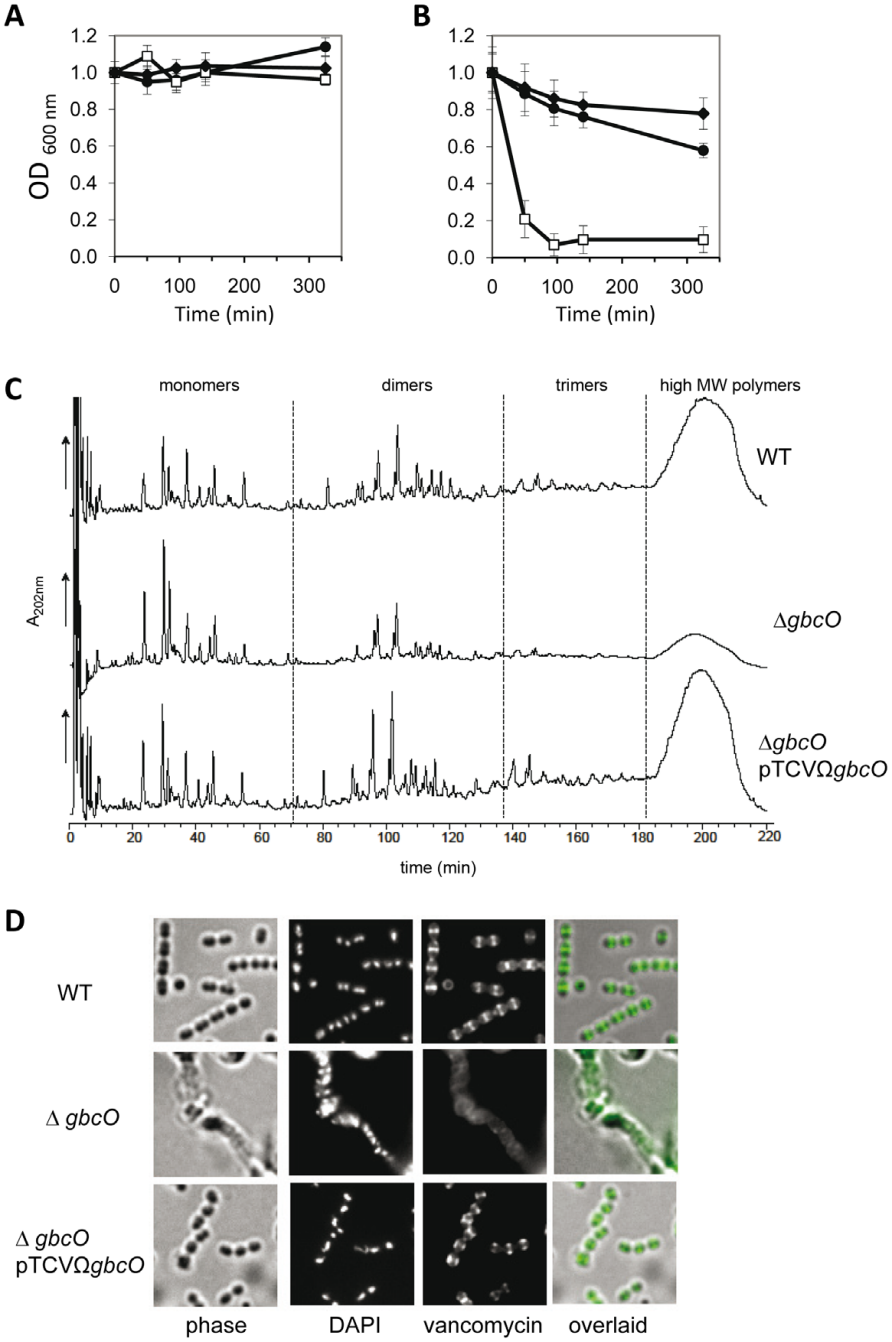
Alterations of the murein sacculus properties and of the PG synthesis in GBC-depleted cells. (**A,B**) Cell lysis assays: exponentially growing cells were harvested and resuspended in PBS buffer (OD_600 nm_ = 1) containing (**A**) 1 mg/ml lysozyme or (**B**) 20 units/ml mutanolysin. Lysis of NEM316 WT (black circles), *ΔgbcO* mutant (white squares), and Δ*gbcO*pTCVΩ*gbcO* complemented (black diamonds) strains was recorded spectrophotometrically at 600 nm. Error bars represent ± S.E. of three independent experiments (**C**) Comparative analysis of the muropeptides resulting from mutanolysin-digested peptidoglycan (see **Text S1**) of NEM316 WT (upper panel), *ΔgbcO* (median panel), and complemented (bottom panel) strains. Muropeptides were separated by RP-HPLC and the peaks were collected and analyzed by MALDI-TOF. (**D**) Fluorescent vancomycin staining of exponentially growing NEM316 WT, *ΔgbcO* mutant and complemented strains. Fluorescent vancomycin (2 mg/ml) was added to exponential-phase cultures for 10 min at 37°C. Cells were harvested, transferred to glass slides, fixed, and observed by fluorescent microscopy as described in Supporting Materials and Methods (see **Text S1**).

In line with this last hypothesis, we consistently observed that the GBS *ΔgbcO* mutant was more susceptible to mutanolysin-induced lysis than WT cells whereas its sensitivity to lysozyme was not affected (**Figure 7A and 7B**). To test further this hypothesis, RP-HPLC separation of the PG-derived muropeptides from WT, *ΔgbcO* mutant, and complemented strains was carried out. More than 50 peaks were analyzed by MALDI-TOF mass spectrometry to deduce the structure of the separated muropeptides (**Figure 7C**). The chromatograms revealed that while the monomeric forms of PG were more abundant in extracts from the mutant strain as compared to the WT (**Figure 7C** **middle row**), the amount of the remaining categories (dimers, trimers, and unresolved high MW oligomers) was lower (**Figure 7C** **upper row**). Quantitative analysis of the chromatograms confirmed this observation and revealed that the cross-linking index plummeted from 34% (WT and complemented strains) to 24% (*ΔgbcO* mutant) (see **Table S3** in supporting information file **Text S1**). The highly cross-linked PG components accumulated in an unresolved peak eluting between 180–220 min. As the area of the peak was strongly reduced in the *ΔgbcO* mutant, the decrease of the PG cross-linking was likely underestimated.

To substantiate the hypothesis of an abnormal incorporation of PG precursors in the cell wall of the *ΔgbcO* mutant, exponentially growing bacteria were stained with fluorescent vancomycin to label the active zone of PG synthesis [30], . As already observed in *S. pneumoniae* R6 [31], vancomycin staining was confined mainly to equatorial and septal regions of NEM316 WT and complemented cells (**Figure 7D** **and Figure S4 upper and lower rows**). In contrast, we observed a low intensity uniform staining over the entire surface of *ΔgbcO* cells (**Figure 7D** **and Figure S4 median rows**). The lack of EqR (**see** **Figure 6C**) together with the disappearance of the discrete vancomycin labeling indicated that, in the absence of GBC, the cell wall biosynthesis machinery was not properly located leading to a dispersed mode of PG synthesis and to the PG cross-linking defect.

### GBC deprivation leads to a mislocalization of the putative PG hydrolase PcsB

**Figure 8:**
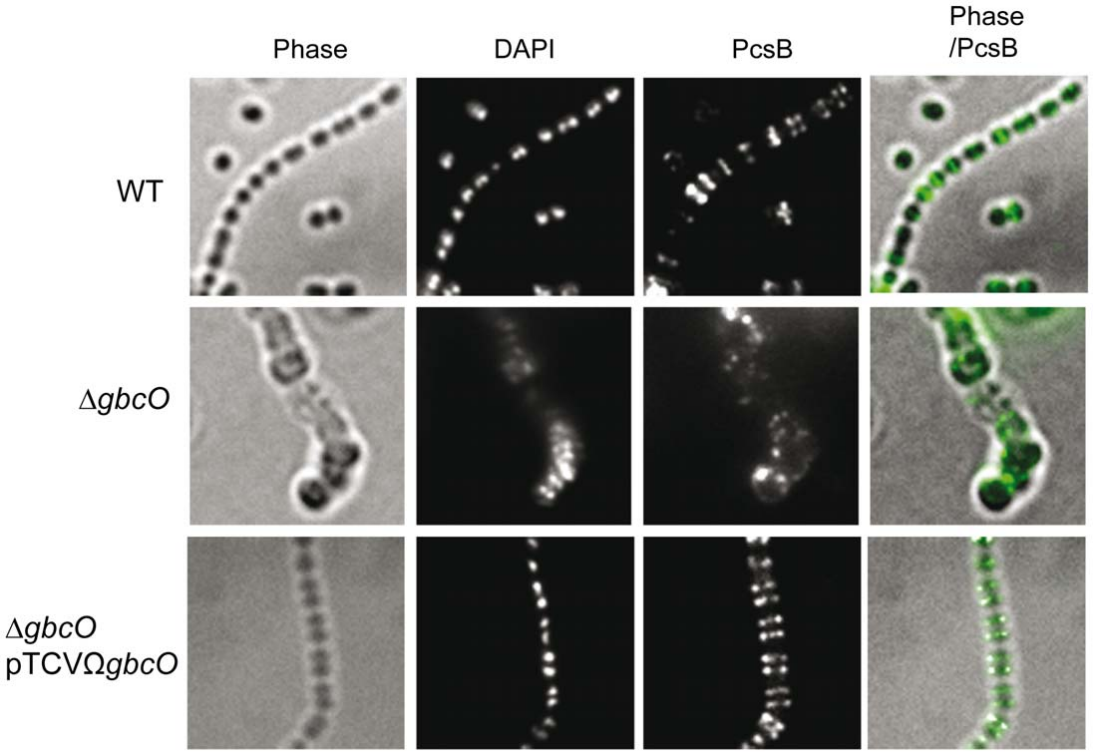
Fluorescent immunolocalization of the putative peptidoglycan hydrolase PcsB. Exponentially growing NEM316 WT, *ΔgbcO* mutant and Δ*gbcO*pTCVΩ*gbcO* complemented strains were harvested, transferred to glass slide, and fixed. IFM with anti-PcsB serum and DAPI staining were performed as described in Materials and Methods.

The consequences of the inactivation of the *gbcO* gene on cell morphology and division were reminiscent to those observed for a PcsB-null mutant of *S. agalactiae* strain 6313 [32], [33]. PcsB (protein required for cell wall separation) is a cell surface located putative PG hydrolase that has orthologs in all species of the Streptococcaceae family. This protein possesses a cysteine-histidine-dependent-amidohydrolase-peptidase (CHAP) domain and is required for proper cell wall synthesis and efficient cell separation in *S. agalactiae* and other streptococci [31], [33]–[35], suggesting that it is involved in PG remodeling. We therefore hypothesized that GBC deprivation could impact the localization of PcsB and the associated PG hydrolase activity. To test this hypothesis, IFM experiments were performed to localize PcsB on the surface of bacterial cells harvested in the exponential phase of growth (**Figure 8**). A PcsB-specific signal located in the equatorial zone of dividing cells corresponding to the site of active PG synthesis was observed in NEM316 WT and complemented strains. This localization was recently observed for the orthologous PcsB protein in *Streptococcus pneumonie* [36]. By contrast, no regular labeling pattern can be distinguished in *ΔgbcO* mutant and PcsB-associated signals were unevenly distributed on the cell surface and in some instances accumulated in foci. This result indicates that GBC is involved in the proper localization of PcsB, a cell wall protein involved in bacterial division and PG biosynthesis.

These results can be paralleled to those recently reported In *B. anthracis*. In this species, a *tagO-like* gene is implicated in the linkage of the pyruvylated cell wall polysaccharide (SCWP) to the envelope and the tunicamycin treatment of *B. anthracis* cultures alters the cell morphology and delocalizes the PG synthesis [25]. This phenotype can be correlated with a mislocalization of BslO, an autolysin involved in cell separation, and whose cell wall localization is SCWP-dependent [37].

### Concluding remarks

The cell wall of *S. agalactiae* contains two PG anchored polysaccharides: the capsule, a major GBS virulence factor [38]–[40], and the GBC, for which we reveal an important biological role linked to PG biosynthesis and cell division. Unencapsulated *S. agalactiae* mutants could be easily constructed *in vitro* and they did not display any growth or morphological defects while being severely affected for virulence in the mouse model [40]. On the other hand, GBC appeared to be pivotal to the *S. agalactiae* cell wall organization and its absence was associated with a substantial loss of fitness. In this study, we provide the first genetic evidence that the synthesis of GBC is initiated by the transfer of GlcNAc phosphate to a lipid phosphate carrier through the activity of GbcO, a close homolog of the enzymes (TagO/TarO) catalyzing the first step of WTA synthesis in *B. subtilis* or *S. aureus* (**Figure 1B**). The growth, morphological, and division defects of the GbcO-null *S. agalactiae* mutant were reminiscent of those reported in WTA-depleted *B. subtilis* [41]–[43] and *S. aureus* [24]. Furthermore, the decrease in PG cross-linking measured in NEM2772 (Δ*gbcO*) strain was also recently observed in *S. aureus* TarO-null mutants [12], [13]. In *S. aureus*, WTA depletion was associated with the delocalization of two proteins involved in PG metabolism: the penicillin-binding protein PBP4 involved in transpeptidation reaction [13] and the autolysin Atl [12]. Similarly, we demonstrate that GBC-depletion caused the mislocalization of PcsB, an important cell-wall enzyme (**Figure 8**). GBC can thus be considered as a functional equivalent of the conventional WTA's found in *B. subtilis* or *S. aureus*. Although the branched rhamnose-rich polysaccharidic structure of GBC is totally different from that of pAdoP-WTA present in *B. subtilis* and *S. aureus*, these polymers share two important properties: first, they both display an anionic character conferred by their high phosphate content; second, they are covalently anchored to the PG which suggests a tight coordination of their synthesis with that of PG. Importantly, the fitness of GBC-depleted mutant was dramatically reduced *in vitro* and we observed that the fitness of the WT strain was similarly reduced when GbcO was inhibited with tunicamycin. These findings indicate that the GBC biosynthesis pathway might constitute a valuable target for the development of novel antibiotics.

The precise structure of Lancefield antigens has been determined for GBC only. However, immunochemical and compositional analysis of Group A, C, E, and G cell wall glycopolymers have similarly revealed a high-rhamnose content [44]–[48]. In these streptococcal species, the biosynthetic pathways and biological roles of the cell wall glycopolymers have not been investigated yet. A careful examination of the genomes of species representative of the major streptococcal phylogenetic lineages (the so-called pyogenic, bovis, salivarius, mutans, mitis, and anginosus groups) revealed the presence of a *gbcO* ortholog together with loci thought to be involved in the synthesis of a rhamnose-containing exopolysaccharide, while orthologs of conventional WTA synthesizing genes were not detected. This analysis suggests that PG-anchored rhamnose-rich polysaccharides are widespread among streptococci including important streptococcal human pathogens (like *S. agalactiae* and *S. pyogenes*). It is likely that their function is similar to that of GBC and inactivation of the streptococcal *gbcO* orthologs constitutes a simple and straightforward approach to validate this hypothesis.

## Materials and Methods

### Bacterial strains, media and growth conditions

The bacterial strains used are listed in **Table S1** in **Text S1**. *S. agalactiae* NEM316 is a ST-23 serotype III strain whose genome has been sequenced [10]. *S. agalactiae* was cultured in Todd-Hewitt (TH) broth (standing filled flasks) or agar (Difco Laboratories, Detroit, MI) at 37°C. *Escherichia coli* DH5α (Invitrogen) used for cloning experiments was grown in Luria-Bertani (LB) medium. Erythromycin was used at 150 µg/ml for *E. coli* and 10 µg/ml for *S. agalactiae*. *E. coli* strains were grown in Luria-Bertani (LB)-broth or on LB-agar. All incubations were at 37°C. Kanamycin was used at concentrations of 20 µg/ml and 500 µg/ml for *E. coli* and *S. agalactiae*, respectively. Gram staining was performed using the bioMérieux Gram Stain kit according to the manufacturer's instructions.

### GBC and PcsB detection by immunofluorescent microscopy (IFM) assays

Cultures harvested in exponential (OD_600_ = 0.5) or stationary phase (6-hours cultures, OD_600_ = 1.5) as indicated in legends to figures were resuspended in PBS at OD_600_ = 1 after three PBS washes. The bacterial suspension (50 µl) was applied on polylysine-coated glass coverslips for 5 min at room temperature, washed twice with PBS and fixed for 15 min with 3% paraformaldehyde. For GBC detection, bacterial cells were harvested in stationary phase and incubated in PBS-BSA 3% with anti-GBC rabbit serum (1/1000) for 30 min. For PcsB detection, bacterial cells were harvested in exponential phase and incubated with mouse serum raised against PcsB (1∶100) obtained as described in **Text S1**. After three PBS washes, the coverslips were incubated with Alexa Fluor A488-conjugated goat anti-rabbit (GBC) or goat anti-rabbit (PcsB) immunoglobulin G (IgG) (Invitrogen) (1/5000) and DAPI (Invitrogen) (1/5000).

### GBC immunodetection after SDS-PAGE and Nitrocellulose transfer

Mutanolysin cell wall extracts were prepared from stationary phase cultures. Bacterial pellet were washed twice in PBS and resuspended at a final OD_600 nm_ = 100 in 50 mM Tris-HCl (pH 7.5) containing sucrose (1 M), mutanolysin (200 U/ml) (Sigma), and incubated for 60 min at 37°C. The suspension was then centrifuged at 5,000 *g* for 15 min at 4°C. The supernatant corresponding to the cell wall extract was collected. To improve the resolution of the GBC band, the cell wall extracts were treated with pronase at 2 mg/ml for 90 min at 60°C. Twenty microliters (∼2 OD units) were loaded on 12% SDS-PAGE for electrophoresis. Transfer was performed on nitrocellulose membrane in a semi-dry electrophoretic transfer cell (Bio-Rad) at 20 V for 20 min in 48 mM Tris, 39 mM glycine, 20% Ethanol, pH 9.2. Membrane was incubated with anti-GBC serum (1/1000) for one hour and then with AlexaA488-conjugated Goat-anti-Rabbit IgG (1/10,000). Membranes were scanned on a Fuji FLA-3000 fluorescent imaging system.

### Vancomycin staining

For staining, a 1∶1 mixture of vancomycin and BODIPY FL vancomycin (Invitrogen) at a final concentration of 2 mg/ml was added to exponentially growing *S. agalactiae* cultures for 10 min, as described [31]. Bacteria were harvested and washed three times with PBS and then resuspended in PBS at OD_600_ = 1. The bacterial suspension was then applied on coverslips and treated as described above.

### Growth conditions for electron microscopy samples

Overnight cultures of *S. agalactiae* were diluted (1/100) into TH and cultivated at 37°C until OD_600 nm_≈0.5. Samples were then prepared for TEM, SEM, and IEM as described in supporting information file **Text S1**.

### Bacterial lysis assays

Bacteria were harvested in exponential phase (OD_600_ = 0.5) and washed twice with PBS. Cells were resuspended at OD_600_ = 1 in PBS and incubated at 37°C in the presence of lysozyme (1 mg/ml) or mutanolysin (20 units/ml). Lysis was followed by changes in optical density at 600 nm at the indicated times.

### Growth curves in the presence of tunicamycin

Growth of *S. agalactiae* strains in the presence of tunicamycin (Sigma) was assayed in TH at 37°C at the following final concentrations: 0, 0.1, 0.25, 0.5, and 2 µg/ml. Growth was recorded in triplicates in 96-well plates in a TECAN M200 plate reader at 600 nm. Maximal growth rates were calculated and reported on the graph as a percentage of the growth rate in absence of tunicamycin.

### Extraction and native PAGE analysis of *S. aureus* WTA

The preparation of cell wall insoluble material by the SDS-boiling procedure, followed by proteolytic digestion and base-catalyzed (NaOH) WTA cleavage was essentially performed as described [25] except that proteinase K was replaced by pronase (2 mg/ml). The native PAGE analysis on T20%C6% gel system was run as described [25]. The WTA bands were stained with the alcyan blue-silver staining protocol using the Bio-Rad silver staining kit as described [25].

### Composition analysis of cell walls by gas chromatography coupled to mass spectrometry

Cell walls from stationary phase cultures were prepared as described for *L. lactis* [49] without HF and TCA treatments to preserve cell wall anchored polysaccharides. Hydrolysis of cell wall samples was performed by treatment in 4 M TFA at 110°C for 3 h, in the presence of xylose added as an internal standard. After drying, the products of the TFA hydrolysis were resuspended in pyridine and derivatized with N-methyl-N-(trimethyl-silyl) trifluoroacetamide (MSTFA) for 30 min at 25°C. The samples were then analyzed by gas chromatography coupled to mass spectrometry (GC-MS) with an Agilent system (GC 6890+ and MS 5973 N, Agilent Technologies). Samples were injected with an automatic injector (Gerstel PAL). Gas chromatography was performed on a 30 m ZB-50 column with 0.25 mm inner diameter and 0.25 µm film thicknesses (Phenomenex). Helium was used as the carrier gas and set at a constant flow rate of 1.5 ml/min. The temperature program was 5 min isothermal heating at 80°C, followed by a 20°C/min oven temperature ramp to 300°C, and a final 3 min heating at 300°C. Compounds were identified by both their retention time and comparison of their electron ionization mass spectra profiles with those of the NIST 05 Mass spectral library (Scientific Instrument Services, Ringoes, NJ, USA). The quantification was done using an external standard calibration curves for each molecule (2.5–25 nmol injected) established with the peak area of specific ion and expressed in nmol/mg cell walls. For purpose of clarity, the results were expressed for each compound as percentage of wild-type values (**Figure 2B**). The average NEM316 wild-type values were: 512.8 nmol/mg for phosphate, 450.5 nmol/mg for rhamnose and 215.7 nmol/mg for muramic acid.

### Purification and structural analysis of PG

*S. agalactiae* cell walls were prepared from exponential phase cultures (OD_600_ = 0.5) as described previously for *Lactococcus lactis* [49] and *Lactobacillus plantarum* [50] with the following modification: an acidic treatment with 5% TCA was performed (24 h at 4°C) before the hydrofluoric acid treatment to remove capsular polysaccharide [17], [51].

Purified PG was digested with mutanolysin (300 U/mg PG) from *Streptomyces globisporus* (Sigma-Aldrich) and the resulting muropeptides were analyzed after NaBH_4_ reduction by RP-HPLC and MALDI-TOF mass spectrometry, as previously reported [52]. Fractions were collected and 1 µl of those containing the main peaks were analyzed by MALDI-TOF mass spectrometry with a Voyager DE STR mass spectrometer (Applied Biosystems) and α-cyano-4-hydroxycinnamic acid matrix. The PG structure of *S. agalactiae* belongs to the A3a group with L-Ala-D-iGln-L-Lys-D-Ala-D-Ala as stem peptide and the dipeptides L-Ala-L-Ala or L-Ala-L-Ser as interpeptide bridges connecting the L-Lys of one stem peptide to the D-Ala in position 4 of the neighbouring subunit [53]. The theoretical masses of the muropeptides with the possible expected structural variations were calculated. The masses determined by Maldi-Tof were then compared with the theoretical masses to classify the HPLC-separated muropeptides as monomers (m/z<1568), dimers (1803<m/z<2647) or trimers (>2647). Cross-linking index (CI) was calculated with the formula according to Glauner (1988) [54]: CI = (1/2 Σdimers+2/3 Σtrimers)/Σ all muropeptides.

## Supporting Information

Figure S1 **Multiple sequence aligment of putative UDP-N-GlcNAc:undecaprenyl-phosphate (UndP) GlcNAc-1-phosphate transferases.** The Swiss-Prot proteins were TagO from *B. subtlis* 168 (O34753), TarO from *S. aureus* N315 (Q7A6R9) and their orthologs GbcO (Gbs0136) from *S. agalactiae* NEM316 (Q8E7L8), RgpG from *S. pyogenes* SF370/M1 (Q9A1G6), and RgpG from *S. mutans* UA159 (Q8CZF2). Black and grey boxes with white letter: 100% and 80% of sequence identity, respectively; grey box with black letter, 60% of sequence identity.
(TIF)

Figure S2 **Morphological aberrations induced by tunicamycin.** *S. agalactiae* NEM316 WT strain (upper row) was cultivated in TH at 37°C with the indicated concentrations of tunicamycin. Light microscopy imaging was performed on living cells in stationary phase. Lower row displays an image of NEM2772 (Δ*gbcO*) that highlights the morphological similarities with tunicamycin-cultivated WT cells. Scale bar: 1 µm.
(TIF)

Figure S3 **Scanning electron microscopy views of** ***S. agalactiae*** **NEM316 WT, Δ** ***gbcO*** **mutant and complemented strains.** Bacteria were cultivated in TH at 37°C and harvested in exponential phase (OD_600 nm_ = 0.5). Different fields at three different scales (see scale bars) revealed that the Δ*gbcO* mutant no longer display typical chains of ovococci.
(TIF)

Figure S4 **Fluorescent vancomycin staining.** Phase contrast and epifluorescence imaging of exponentially growing bacteria labeled with DAPI (DNA marker) and fluorescent vancomycin (marker of PG synthesis sites) as indicated in Supporting Materials and Methods (see **Text S1**). (Scale bars 1 mm). The regular vancomycin labeling is lost in Δ*gbcO* strain.
(TIF)

Text S1 **Contains supporting** **materials and methods** **; table S1, table S2 and table S3 and supporting references.**
(DOCX)

Video S1 **Growth of NEM316 WT was performed at 37°C. The elapsed time is 4.5 hours.**
(AVI)

Video S2 **Growth of NEM316** ***ΔgbcO*** **mutant was performed at 37°C. The elapsed time is 6 hours.**
(AVI)
