# Supplementary figures and images for: Role of the Group B Antigen of Streptococcus agalactiae: A Peptidoglycan-Anchored Polysaccharide Involved in Cell Wall Biogenesis
Source: PLoS Pathog. 2012 Jun 14;8(6):e1002756. doi: 10.1371/journal.ppat.1002756 (PMC3375309; doi:10.1371/journal.ppat.1002756)

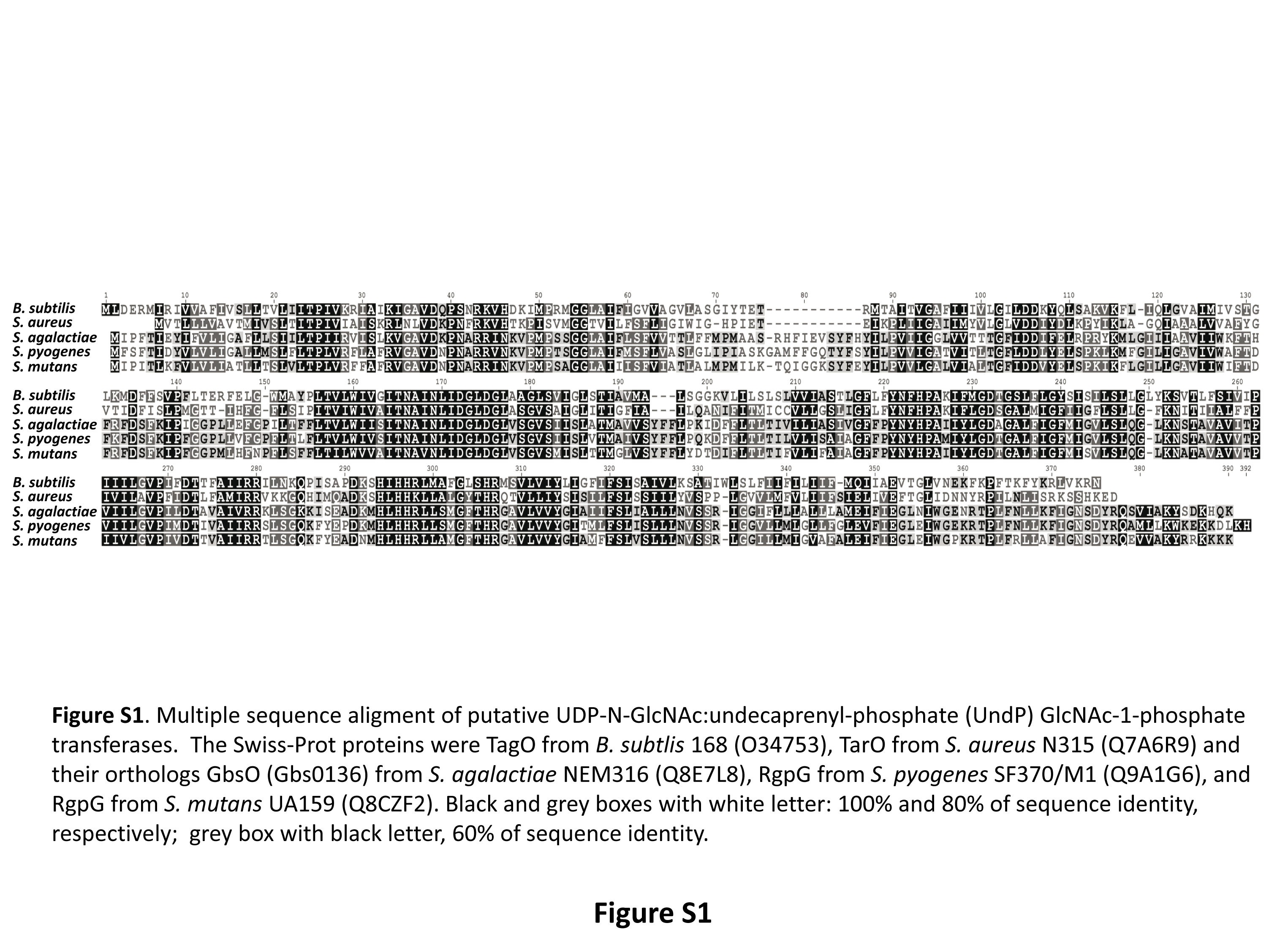

Supplement: Figure S1 — Multiple sequence aligment of putative UDP-N-GlcNAc:undecaprenyl-phosphate (UndP) GlcNAc-1-phosphate transferases. The Swiss-Prot proteins were TagO from B. subtlis 168 (O34753), TarO from S. aureus N315 (Q7A6R9) and their orthologs GbcO (Gbs0136) from S. agalactiae NEM316 (Q8E7L8), RgpG from S. pyogenes SF370/M1 (Q9A1G6), and RgpG from S. mutans UA159 (Q8CZF2). Black and grey boxes with white letter: 100% and 80% of sequence identity, respectively; grey box with black letter, 60% of sequence identity. (TIF) [file ppat.1002756.s001.tif]

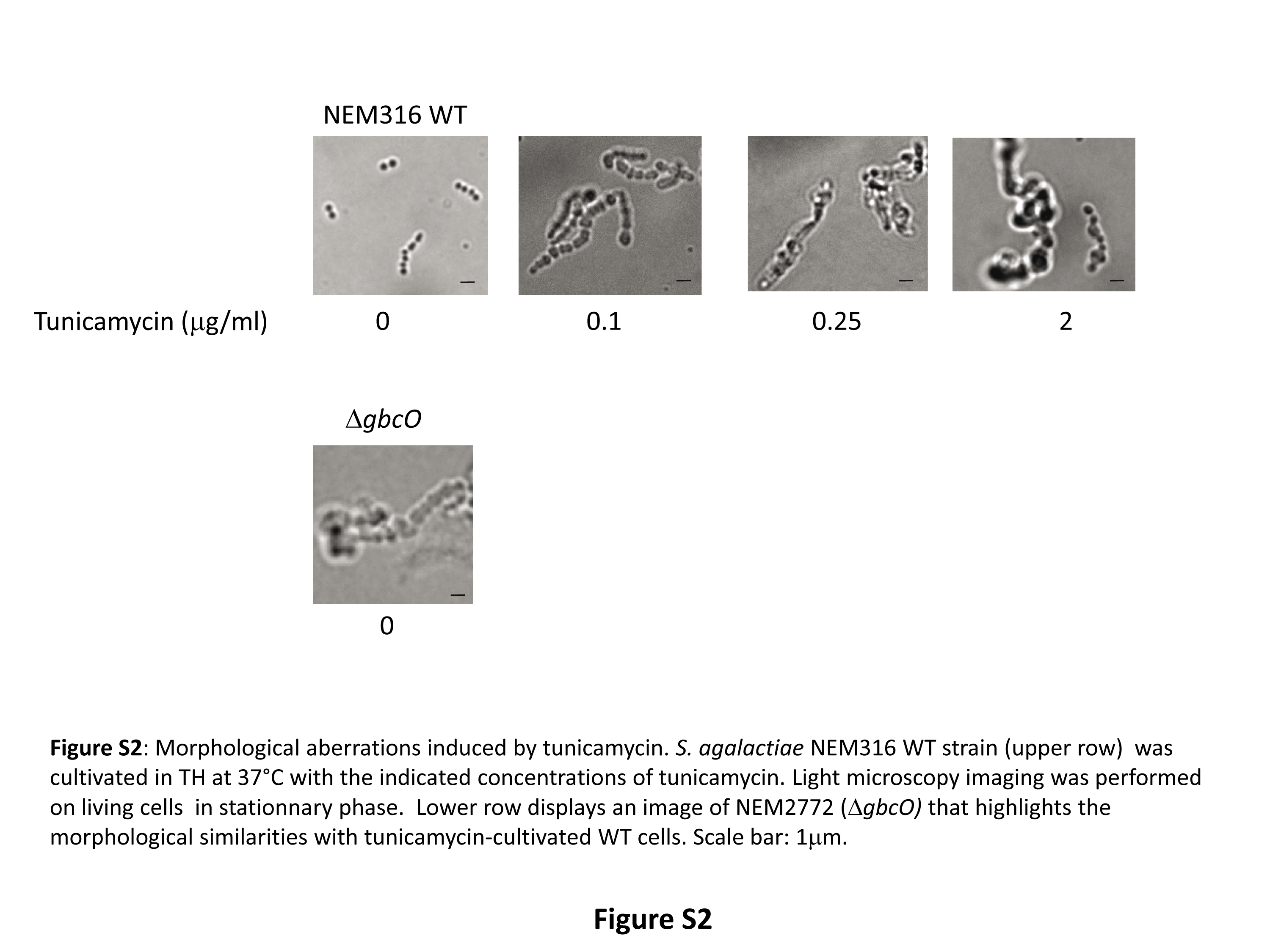

Supplement: Figure S2 — Morphological aberrations induced by tunicamycin. S. agalactiae NEM316 WT strain (upper row) was cultivated in TH at 37°C with the indicated concentrations of tunicamycin. Light microscopy imaging was performed on living cells in stationary phase. Lower row displays an image of NEM2772 (ΔgbcO) that highlights the morphological similarities with tunicamycin-cultivated WT cells. Scale bar: 1 µm. (TIF) [file ppat.1002756.s002.tif]

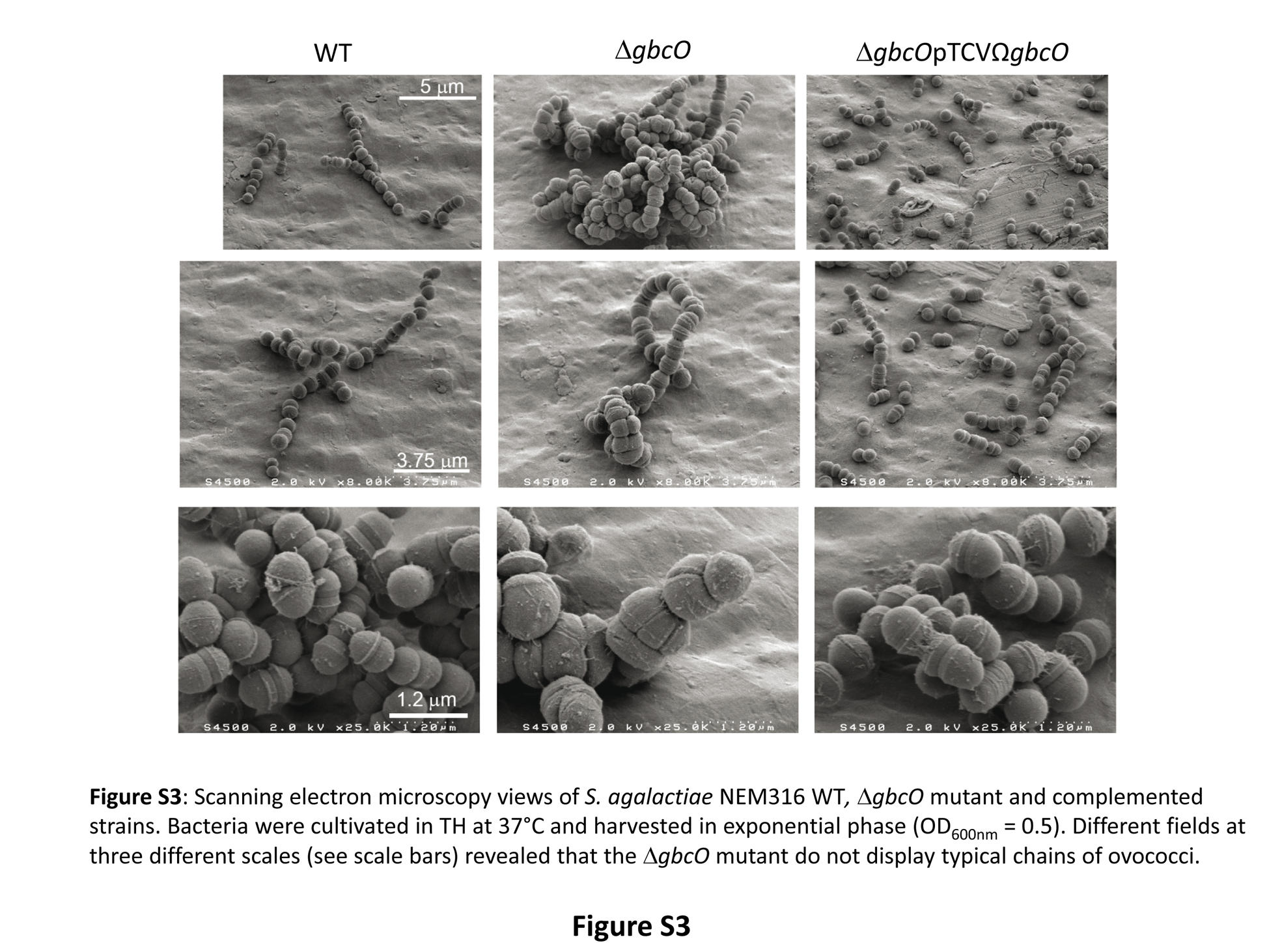

Supplement: Figure S3 — Scanning electron microscopy views of S. agalactiae NEM316 WT, Δ gbcO mutant and complemented strains. Bacteria were cultivated in TH at 37°C and harvested in exponential phase (OD600 nm = 0.5). Different fields at three different scales (see scale bars) revealed that the ΔgbcO mutant no longer display typical chains of ovococci. (TIF) [file ppat.1002756.s003.tif]

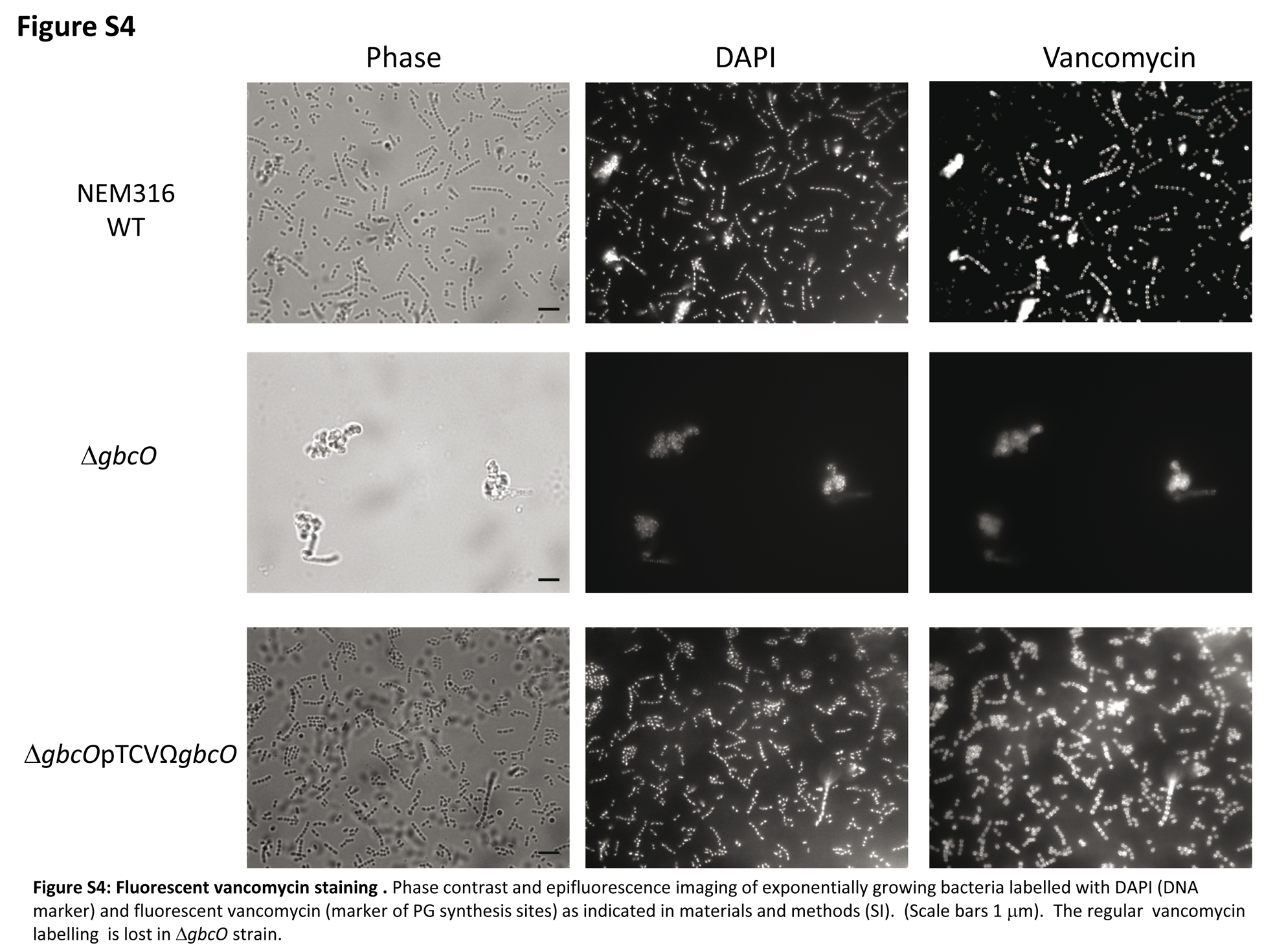

Supplement: Figure S4 — Fluorescent vancomycin staining. Phase contrast and epifluorescence imaging of exponentially growing bacteria labeled with DAPI (DNA marker) and fluorescent vancomycin (marker of PG synthesis sites) as indicated in Supporting Materials and Methods (see Text S1). (Scale bars 1 mm). The regular vancomycin labeling is lost in ΔgbcO strain. (TIF) [file ppat.1002756.s004.tif]
