# Supplementary material for: Role of the Group B Antigen of Streptococcus agalactiae: A Peptidoglycan-Anchored Polysaccharide Involved in Cell Wall Biogenesis
Source: PLoS Pathog. 2012 Jun 14;8(6):e1002756. doi: 10.1371/journal.ppat.1002756 (PMC3375309; doi:10.1371/journal.ppat.1002756)
Supplement: Text S1 — Contains supporting materials and methods ; table S1, table S2 and table S3 and supporting references. (DOCX) [file ppat.1002756.s005.docx]

# ****TEXT S1 SUPPORTING INFORMATIONS****

# ****SUPPORTING MATERIALS AND METHODS****

## General DNA techniques

Standard recombinant techniques were used for nucleic acid cloning and restriction analysis (1). Plasmid DNA from *E. coli* was prepared by rapid alkaline lysis using the QIAprep Spin Miniprep Kit (Qiagen). Genomic DNA from *S. agalactiae* was prepared using the DNeasy Blood and Tissue Kit (Qiagen). PCR was carried out with Phusion Taq polymerase as described by the manufacturer (Finnzymes).

**Genetic manipulations**

The primers used for the construction of mutant are listed in Table S2 (Text S1, see below). To construct an *S. agalactiae* *gbcO* mutant (NEM2772), 937 bp of the 1161-bp long *gbcO* gene were replaced by the promoterless and terminatorless kanamycin resistance cassette *aphA-3*(2) inserted in the same direction of transcription. This was done by ligating, after digestion with the appropriate enzymes, the following amplicons: O1-O2 (5' end of *gbcO*), KanK-KanB (*aphA-3* gene), and O3-O4 (3' end of *gbcO*). The corresponding *Eco*RI-*Pst*I fragment was cloned into the thermosensitive shuttle plasmid pG1 and the resulting recombinant vector, pG1Ω*gbcO*∆, was introduced by electroporation into NEM316. The

S. agalactiae gbcO mutant was screened and characterized by DNA sequencing with the primers O5 and O6 as described (3). For complementation analysis, the *gbcO* gene was amplified with primers O7-O8, digested with the appropriate enzymes, and ligated into the shuttle expression vector pTCV-ermΩP*tet* (4) to give pTCV-ermΩP*tet–gbcO* (referred to as pTCVΩ*gbcO* throughout the manuscript). This vector was introduced by electroporation in the WT (*S. agalactiae* NEM316 and *S. aureus* RN4220) and mutant (NEM316 ∆*gbcO* and RN4220 ∆*tarO*) strains.

## Time-lapse microscopy experiments

Overnight culture of bacterial strains were deposited on the CoverWell incubation chamber (Grace Bio-Labs), overlaid with 150 µl of THY medium containing 0.03 % of agar (Difco) and covered with glass slide. Temperature was maintained at 37°C and bacterial growth was followed by taking images each 30 seconds during 18 hours with an Axiover 40 CFL microscope (objective 40X, Carl Zeiss Microlmaging GmbH). Images were captured with axiovision software and processed with ImageJ (v. 1.44p).

## Immunofluorescence imaging

Slides were examined with a Leica DMRA2 microscope equipped with a
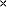
100 magnification oil-immersion objective and a COOLSNAP HQ camera (Roper Scientific, USA). Images were captured and processed with METAMORPH V6.3r5. The Figures were prepared with Adobe Photoshop CS5.

**Production of anti-PcsB mouse serum**

The 5'-moiety of the NEM316 GBS *pcsB* gene (*gbs0016*) was PCR amplified with the primers O9 and O10 and cloned into the pET28-16 expression vector (5). The resulting truncated 272-aa PcsB protein with a COOH-terminal His tag was produced in *E. coli* BL21 (λDE3) strain and purified on Nickel resin as described (www.qiagen.com/goto/Ni-NTA). The purified protein was injected 4 times (10 µg/injection) over a 45-day period in BalB/C mice for the generation of pAbs. Mice serum were collected three weeks after the fourth injection.

## Transmission electron microscopy (TEM) experiments

Pellets were fixed for 3 hours at RT with 2% glutaraldehyde in 0.1 M Na cacodylate buffer pH 7.2. Samples were then post fixed with 1% osmium tetroxide containing 1.5% potassium cyanoferrate and 2% uranyl acetate, dehydrated in ethanol (30% to 100%), and substituted gradually in mix of propylene oxyde-epon and embedded in Epon. Thin sections (70 nm) were collected onto 200 mesh cooper grids, and counterstained with lead citrate. Grids were examined with Zeiss EM902 electron microscope operated at 80kV (Carl Zeiss – France). Images were acquired with a charge-coupled device camera (Megaview III) and analysed with ITEM Software (Eloïse, France) MIMA2 Platform, INRA-CRJ ([http//MIMA2@jouy.inra.fr](mailto:http//MIMA2@jouy.inra.fr)).

## Scanning electron-microscopy (SEM) sample preparation

The samples for scanning electron microscopy (SEM) were immersed in a fixative solution containing 3% glyceraldehyde for 2 h and put onto sterile glass microfibers filters (GF/G 24 mm, Whatman, Fontenay sous Bois, France). The samples were air-dried and then coated with palladium for 210s at 800V and 10 mA. Imaging of cells was performed with a scanning electron microscope Hitachi S-4500. SEM imaging was performed by Thierry Meylheuc on the MIMA2 imaging platform (INRA, MICALIS, Massy-Palaiseau).

## Immunoelectron microscopy (IEM)

The subcellular location of GBC was analyzed using IEM on thin sections (<100 nm) of frozen cells with a sandwich labelling technique employing colloidal gold complexes. The bacteria were fixed into a solution containing 1X PHEM buffer (pH 6.9)-4% (wt/vol) paraformaldehyde-0.25% (wt/vol) glutaraldehyde for 2 h at room temperature. The bacteria were pelleted, resuspended in fixation solution containing 0.4% paraformaldehyde, and kept at 4°C overnight. The fixed cells were then embedded in 12% (wt/vol) gelatin (15), and ultrathin cryosections were cut at -120°C and processed for immunolabeling.

All incubations, washings, and staining steps were performed at room temperature. Grids were blocked with PBS-Glycine 50mM, followed with PBS-1% BSA, 10% normal goat serum (Sigma, France). Antiserum against GBC was added at a 1/1000 dilution in PBS containing 1% BSA and 2% normal goat serum. After 2 h of incubation, the grids were washed twice for 3 min in PBS-1% BSA and then goat anti-rabbit IgG coupled to 10 nm colloidal gold particles (British Biocell International – TEBU, France) was added at 1/50 dilution for 1 hour. After immunolabeling, the sections were postfixed in 0.25% (wt/vol) glutaraldehyde, stained with uranyl acetate, and embedded in mix of 2%methylcellulose- 5% uranyl acetate (4/1) on ice. Grids were examined with a Zeiss EM902 electron microscope operated at 80kV (Carl Zeiss – France). Images were acquired with a charge-coupled device camera (Megaview III) and analysed with ITEM Software (Eloïse, France) MIMA2 Platform, INRA-CRJ ([http//MIMA2@jouy.inra.fr](mailto:http//MIMA2@jouy.inra.fr)).

## **Table S1**

**Bacterial strains and plasmids**

| Strain or plasmid | Relevant properties^a^ | Source or reference |
| --- | --- | --- |
| Strains |  |  |
| *Escherichia coli* |  |  |
| DH5α | *recA1* *gyrA* (Nal), Δ(*lacIZYA-argF*)[Φ80D*lac* Δ(*lacZ*)M15] | Gibco-BRL |
| BL21λDE3 | F^-^ *omp*T *gal* (*dcm*) (*lon*) *hsd*S_B_(r_B_^-^ m_B_^-^) *endA1* *hsdR1*7(r_K_^-^m_K_^+^) | (6) Novagen |
| *Streptococcus agalactiae* |  |  |
| NEM316 | Serotype III, ST-23 strain | (7) |
| NEM2772 | NEM316∆*gbcO* (*gbcO*Ω*aphA-3*; Km) | This work |
| Plasmids |  |  |
| pG1 | Em; *oriR* pUC, *oriR^ts^* pWV01; MCS pUC18 | (8) |
| pET28/16 | Ap, *oriR* pUC, T7 promoter, His-Tag coding sequence | (5) |
| pTCV-ermΩPtet | Em, Km, *oriR* pACYC184, *oriR* pAMβ1, P*tetM* promoter (Tn*916*); MCS pUC18 | (9) |

^a^ Ap, Km, and Em are resistance to ampicillin, kanamycin, and erythromycin, respectively. MCS, multiple cloning site.

## **Table S2**

**Oligonucleotides used in this study**

| Name | Sequence (5' to 3')^a^ |
| --- | --- |
| O1 | GATACGAATTCCCATGCCAAATTAGCAGA |
| O2 | GTGCGGTACCCGGTTTATCAACCGCTCCTACCT |
| O3 | CGGGGGATCCGGTGAGAATCGGACACCACTAT |
| O4 | GCAAGCTGCAGACCCAAACGAGCACGTTCAT |
| O5 | GAATTAGCAGATTTGGGTGAC |
| O6 | TGGAACCTCTGAAGGGTATTGC |
| O7 | TTCAAGGATCCAAATGATTGAGATGGGATAAAATGATACC |
| O8 | TTGATCTGCAGTACAGACATTGTACACTCCT |
| O9 | TGATGCCATGGACTCGAAAATTGCTGCTACTG |
| O10 | TAGAAGGATCCATTTCAGTTCTCGGTTGTG |
| KanK | GGGGTACCTTTAAATACTGTAG |
| KanB | TCTGGATCCTAAAACAATTCATCC |

**a The restrictions sites used for cloning were underlined.**

## Table S3

**Percentages of monomer, dimer and trimer muropeptides in peptidoglycan of *S. agalactiae* NEM316 strains and Δ*gbcO* variants.**

| Muropeptides oligomers (%) | WT^b^ | Δ*gbcO*^b^ | Δ*gbcO*pTCV*gbco*^b^ |
| --- | --- | --- | --- |
| Monomer | 36.9 | 55.6 | 35.9 |
| Dimer | 49.4 | 37.2 | 50.6 |
| Trimer | 13.7 | 7.2 | 13.5 |
| >cross-linking index^a^ | 33.8 | 23.4 | 34.3 |

^a^ The cross-linking index was calculated according to (10) with the formula: (1/2 Σ dimers + 2/3 Σ trimers)/Σ all muropeptides.

^b^The percentage of each peak was calculated as the ratio of the peak area relative to the sum of areas of the monomers, dimers, and trimers peaks of the corresponding chromatogram.

# SUPPORTING REFERENCE

1. Sambrook J, Fritsch EF, & Maniatis T (1989) *Molecular Cloning: a Laboratory Manual* (Cold Spring Harbor Laboratory, NY, New-York).

2. Trieu-Cuot P & Courvalin P (1983) Nucleotide sequence of the Streptococcus faecalis plasmid gene encoding the 3'5"-aminoglycoside phosphotransferase type III. *Gene* 23(3):331-341.

3. Lalioui L*, et al.* (2005) The SrtA Sortase of Streptococcus agalactiae is required for cell wall anchoring of proteins containing the LPXTG motif, for adhesion to epithelial cells, and for colonization of the mouse intestine. *Infect Immun* 73(6):3342-3350.

4. Trieu-Cuot P, Carlier C, Poyart-Salmeron C, & Courvalin P (1991) Shuttle vectors containing a multiple cloning site and a lacZ alpha gene for conjugal transfer of DNA from Escherichia coli to gram-positive bacteria. *Gene* 102(1):99-104.

5. Chastanet A, Fert J, & Msadek T (2003) Comparative genomics reveal novel heat shock regulatory mechanisms in Staphylococcus aureus and other Gram-positive bacteria. *Mol Microbiol* 47(4):1061-1073.

6. Studier FW & Moffatt BA (1986) Use of bacteriophage T7 RNA polymerase to direct selective high-level expression of cloned genes. *Journal of Molecular Biology* 189(1):113-130.

7. Glaser P*, et al.* (2002) Genome sequence of Streptococcus agalactiae, a pathogen causing invasive neonatal disease. *Mol Microbiol* 45(6):1499-1513.

8. Mistou MY, Dramsi S, Brega S, Poyart C, & Trieu-Cuot P (2009) Molecular dissection of the secA2 locus of group B Streptococcus reveals that glycosylation of the Srr1 LPXTG protein is required for full virulence. *J Bacteriol* 191(13):4195-4206.

9. Poyart C, Lamy MC, Boumaila C, Fiedler F, & Trieu-Cuot P (2001) Regulation of D-alanyl-lipoteichoic acid biosynthesis in Streptococcus agalactiae involves a novel two-component regulatory system. *J Bacteriol* 183(21):6324-6334.

10. Glauner B (1988) Separation and quantification of muropeptides with high-performance liquid chromatography. *Anal Biochem* 172(2):451-464.
